# Performance of individual dietary diversity score to identify malnutrition among patients living with HIV in Ethiopia

**DOI:** 10.1038/s41598-021-98202-6

**Published:** 2021-09-21

**Authors:** Foziya Mohammed Hussien, Wondwosen Mebratu, Aragaw Yimer Ahmed, Tefera Chane Mekonnen, Anissa Mohammed Hassen, Zinet Abegaz Asfaw, Hamid Yimam Hassen, Kalkidan Hassen Abate

**Affiliations:** 1 Department of Public Health, College of Medicine and Health Science, Wollo University, Dessie, Ethiopia; 2 Department of Internal Medicine, School of Medicine, College of Medicine and Health Science, Wollo University, Dessie, Ethiopia; 3 Department of Public Health, College of Medicine and Health Science, Mizan-Tepi University, Mizan-Tepi, Ethiopia; 4 Department of Public Health Nutrition, School of Public Health, College of Medicine and Health Sciences, Jimma University, Jimma, Ethiopia

**Keywords:** Diseases, Health care, Medical research, Risk factors

## Abstract

There is a lack of uniformity in developing and validating indicators of nutritional status among People Living with Human Immunodeficiency Virus (PLHIV). Experiences from low and middle-income countries are scant, and differences in methodological and analytical approaches affect the comparability and generalizability of findings. Therefore, this study investigated the performance of individual diversity score (IDDS) as a proxy indicator of nutritional status among PLHIV. We conducted a facility-based cross-sectional study among 423 PLHIV who were under Antiretroviral Treatment (ART) at clinics in Bahir-Dar, Ethiopia. We collected data on sociodemographic, dietary, clinical, and anthropometric measures. Dietary intake was assessed using 24-Hour dietary recall. Body Mass Index (BMI) was calculated to assess the nutritional status of study subjects. The receiver operating characteristic (ROC) curve analysis was used to assess the ability of the IDDS and Minimum Dietary Diversity for Women (MDD-W) to detect poor nutritional status. Furthermore, sensitivity, specificity, Predictive Values (PPs), and Likelihood Ratios (LRs) were calculated at different cut-off points. IDDS showed good reliability with Cronbach’s Alpha of 0.76. The Area Under the Curve (AUC) of IDDS was 78.5 (95%CI 73.9–83.4). At the IDDS cut-off of 4, the sensitivity and specificity of IDDS to indicate nutritional status were 88.0% (95%CI 81.0–93.0) and 71.0% (95%CI 66.0–76.0), respectively. The AUC of MDD-W was 74.1%, and at the cut-off of 4 the sensitivity and specificity of MDD-W to indicate undernutrition were 73.0% and 72.0%, respectively. Both IDDS and MDD-W have good accuracy as a proxy indicator for measuring the nutritional status of PLHIV. In the prevention of undernutrition among PLHIV especially in a resource-limited setting, IDDS and MDD-W can be used to assess nutritional status.

## Introduction

Globally, 38.0 million people live with HIV and the disease accounted for 690,000 deaths in 2019^1^. In Ethiopia, an estimated 710, 000 people were living with HIV and there were 20,000 HIV-related deaths^2^. Although more people are surviving due to increased availability of ART, there is still a need to increase survival and quality of life by improving nutrition. Eating a diversified diet for PLHIV increases resistance to opportunistic infections improves energy, and makes a person generally stronger and more productive^3^.

Lack of diversified diets and malnutrition are public health concerns worldwide, particularly in low and middle-income countries (LMICs)^4^. Nutritional guidelines recommend increasing the variety of food as well as food groups consumed emphasizing the absence of a single food that contains all the required nutrients for optimal health^5^. Therefore, dietary diversity is needed to meet the requirements of micronutrients and energy especially for those who are at risk of nutritional deficiencies including PLHIV^6,7^. Besides, a diversified diet can induced immunity then increase antiretroviral tolerance (reduce viral load and side effects), absorption, leading to a decrease in morbidity and improvement in survival time^8–12^.

Accurate and consistent measurement of dietary intake and patterns of eating behavior is necessary to monitor and evaluate the effectiveness of public health interventions aimed at improving diet and reduce malnutrition.[13]Dietary methods are useful tools for nutritional assessment and monitoring of economic conditions^13^. IDDS is an indicator for assessing the quality of an individual's dietary habits^7,14^. It is more of a proxy of the nutrient (mainly micronutrient) adequacy of the diet of an individual^7^. Diversity scores are attractive to use because of their ease of measurement and interpretation. However, there is no international consensus on the number and type of food groups to include in the IDDS, and consistent cutoffs to determine the adequacy of dietary diversity^7,15^. Therefore, in resource-limited settings, a simple, easy to use, and accurate method is needed to assess nutritional status. Tool validation is of particular importance in any dietary assessment method^13,16^.

Dietary diversity scores (DDS) have been validated in different age or sex groups as a proxy measure for the macro or micronutrient adequacy of the diet^8,17–20^. They are associated with the mean micronutrient adequacy of the diet^21,22^. DDS has also been shown to be associated with the nutritional status of individuals after adjusting for socioeconomic factors^23–25^. Despite the relationship between DDS and individual nutritional status, the use of such an indicator for nutritional assessment, monitoring, and evaluation is still controversial^10,26–29^. There is also a lack of consensus on how to measure and operationalize DDS^10,15^. These inconsistencies could be attributable to disparities in sociodemographic characteristics, economic, and food type across contexts^17^. These issues impede the adoption of standardized indicators, which would be useful for comparing dietary diversity across populations and over time^30^.

Due to a lack of uniformity and consensus, research is needed in LMICs to develop valid and reliable indicators of dietary diversity^31,32^. Furthermore, in LMICs measuring dietary diversity in the context of assessing nutritional status is scant, and differences in methodological and analytical approaches affect the comparability and generalizability of findings^15,31^. Our previous study showed that household dietary diversity (HDD) has a good validity to assess nutritional status in PLHIV. However, the performance of individual dietary diversity, a tool that assesses the overall quality of an individual's diet, has yet to be validated.

Furthermore, poor dietary diversity in PLHIV has been associated with weight loss, disease progression opportunistic infections, and poor survival^33^. Thus, adequate dietary diversity is a key to strengthen the immunity system, maintain muscle mass, prevent viral progression and keep PLHIV healthy^33,34^. Therefore, measuring the validity and determining IDDS cutoffs in this population group is important to consider targeted interventions. Hence, we evaluated the performance of individual dietary diversity to identify nutritional status among PLHIV.

## Methods

### Study setting, design, and participants

The study was conducted in Bahir Dar city, the capital of Amhara region in North-western Ethiopia, situated on the southern shore of Lake Tana. The commonly cultivated and consumed foods in the area are *teff,* maize, barley, wheat, tomato, and green leafy vegetables, whereas fruits are rarely consumed in the area^35^. A facility-based, cross-sectional study was conducted among 423 PLHIV who were attending Anti-retroviral treatment (ART) clinics in Bahir Dar, Ethiopia, from January to February 2017. All ART clinics, seven of which were public, and three private, were included in the study. The study included men and women aged 18 years or older who were HIV positive and under treatment at the ART centers during the data collection period. We excluded critically ill patients and pregnant women from the study. The sample size (423) was determined using a single population proportion formula assuming a 50% proportion, with a 95% confidence level, 5% margin of error, and an expected 10% non-response rate. From a total of 10,666 patients, the sample size was allocated to each ART clinic using proportional to population size (PPS). Then, eligible participants were taken from each selected ART clinic consecutively until the required sample size was obtained.

### Data collection technique

We collected data on socioeconomic status including education and occupation, dietary habits, clinical conditions, using a structured questionnaire, and anthropometric measurements. The questionnaire was adapted from the Ethiopian Demographic and Health Survey (EDHS) 2016 and modified accordingly^36^.

### Index variable

Dietary intake data was collected using a 24-h recall method. Additionally, the individual dietary diversity sheet including staple foods and beverages which was grouped into 9 groups: (1) Starchy stables; (2) Vitamin-A rich vegetables and fruits; (3) Other fruits and vegetables; (4) meat and fish; (5) Dark green leafy vegetables; (6) Organ meat; (7) Egg; (8) Legumes, nuts and seeds; (9) Milk and milk product was used to collect the data^7^. After the study subjects were asked to remember whatever they consumed in the past 24 h, the probing questions were followed to recall other food items. All foods eaten by the individual of interest, consumed inside or outside the home, regardless of where the food prepared was included. Very small food quantities less than one teaspoon (< 10 g) were excluded. If a person is on a special occasion such as fasting, funeral, and feast, the next person was selected. A set of 9 food groups were used to guide the scoring as per the food items consumed, with 0 being the minimum score and 9 as the maximum^37^.

The Minimum Dietary Diversity for Women (MDD-W) was grouped into 10 food groups: (1) Grains, roots, and tubers; (2) Pulses (Legumes); (3) Nuts and seeds; (4) Milk and milk products; (5) Meat, poultry and fish; (6) Eggs; (7) Dark green leafy vegetables; (8) Other vitamin A-rich fruits and vegetables; (9) Other vegetables; (10) Other fruits^38^. A set of 10 food groups were used to guide the scoring as per the food items consumed, with 0 being the minimum score and 10 as the maximum^38^.

### Outcome variable

Height was taken in a standing position without wearing shoes, at the apex of the head, with 0.1 cm precision^39^. Similarly, weight was taken by removing footwear and heavy clothing, using digital weighing scales, to the nearest 0.1 kg. Measurements were taken twice and the mean score was recorded^40^. Body mass index (BMI) was calculated to determine an individual's nutritional status by dividing weight in kilograms (kg) by height in meters (m) squared, then classified as underweight (< 18.5 kg/m^2^), healthy weight (18.5–24.9 kg/m^2^), overweight (25–29.9 kg/m^2^), and obese (> 30 kg/m^2^)^41^. Finally, those participants identified as undernutrition were given nutritional counseling and therapeutic feeding in collaboration with the clinicians working at ART clinics.

### Data quality control

To maintain the quality of data, training was given to the data collectors (clinical staff nurses) and supervisors on the data collection, interviewing, and measurement techniques. Then, the collected data were revised and possible errors were returned to the data collectors for correction daily. Moreover, the pre-test was conducted before the actual data collection period. Measuring equipment was calibrated every ten measurements.

### Data analysis

Data were entered into EpiData version 3.1 and exported to a free statistical software R version 4.0.3 for further processing and analysis^42^. The characteristics of study participants were summarized using descriptive statistics including mean/median with standard deviation (SD)/interquartile range (IQR), and absolute and relative frequencies.

For all items of IDDS, reliability analysis was done and Cronbach’s Alpha coefficient was calculated. A Cronbach’s Alpha value of > = 0.9, 0.7–0.9, 0.5–0.7, 0.3–0.5, respectively were considered as very high, high, moderate, and low reliability^43^.

To assess the ability of the IDDS to detect poor nutritional status, we used Receiver operating characteristic (ROC) curve. In a ROC curve, the true positive rate was plotted as a function of the false positive rate at different cut-off points of the test variable (number of scores) in comparison with BMI as a reference standard. Each point on a ROC curve represents a sensitivity/specificity pair corresponding to a particular decision threshold. The optimal cut-off point was also determined by maximizing the Youden’s index (J = Sensitivity + Specificity − 1) and, with the minimum distance to the upper left corner on the Receiver operating characteristic (ROC) curve.

The area under the ROC curve (AUC) was used as an indication of the predictive power or the accuracy of the proxy indicator to correctly classify the nutritional status. A perfect classification by the proxy indicator would result in an AUC of 1. AUC below 0.6 is considered not acceptable^44^. Furthermore, the discriminatory and predictive potential of IDDS against BMI was assessed using sensitivity, specificity, positive predictive value (PPV), negative predictive value (NPV), positive and negative likelihood ratio with 95% confidence interval.

### Ethical consideration

Ethical approval was obtained from the Institutional Review Board of Wollo University, College of Medicine and Health sciences. Written informed consent was obtained from each participant after an explanation of the study purpose, description of the possible risks and benefits. Privacy and confidentiality of the collected information were ensured throughout the process. We confirm that the study was in compliance with the principles of the declaration of Helsinki.

## Results

### Socio-demographic characteristics

A total of 423 adults living with HIV aged 18 years or older were enrolled in the study. Of the respondents 42.6% were male and 60.0% were married. The mean age was 34.8 years (SD 8.4), and 56.3% were within the 18–35 years age group. The average family size was 3 (SD 2) people per household. Sixty-eight (16.1%) of the respondents could not read or write. About 64.1% reported an average monthly income higher than 1001(37.3 USD) Ethiopian birr per month with the median income of 2000 birr (1 USD = 26.84 ETB). Above a quarter (26.2%) were employed in public institutions and 6.1% were rural residents (Table 1).

**Table 1:** Sociodemographic characteristics of the study participants in ART clinics of Bahir Dar, 2017 (N = 413).

Variable	Frequency	Percent
**Sex**		
Male	180	42.6
Female	243	57.4
**Age group (years)**		
18–35	238	56.3
36–55	179	42.3
56+	6	1.4
**Religion**		
Orthodox	331	78.3
Muslim	71	16.8
Protestant	21	5.0
**Ethnicity**		
Amhara	378	89.4
Tigre	24	5.7
Oromo	19	4.5
Others	2	0.5
**Marital status**		
Single	68	16.1
Married	254	60.0
Divorced	75	17.7
Widowed	26	6.1
**Educational status**		
Cannot read and write	68	16.1
Primary education (1–8)	115	27.2
Secondary education (9–12)	126	29.8
Tertiary education (> 12)	114	27.0
**Occupation**		
Government employed	111	26.2
Farmer	7	1.7
Self-employed	78	18.4
Daily laborer	69	16.3
Merchant	76	18.0
Housewife	77	18.2
Retired	5	1.2
**Monthly HH Income**		
< = 500	73	17.3
501–1000	79	18.7
1001+	271	64.1
**Residence**		
Urban	397	93.9
Rural	26	6.1
Total	423	100

### Individual dietary diversity of PLHIV

**Figure 1:**
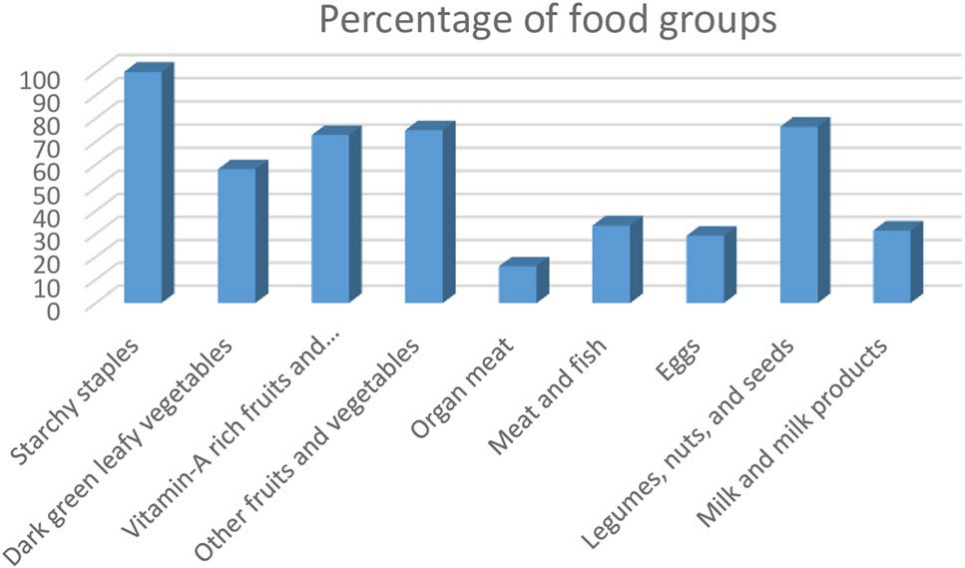
The prevalence of IDD food groups consumed by PLHIV in all ART clinics of Bahir Dar, 2017 (N = 413).

Figure 1 summarizes the intake of food types within 24 h preceding the survey date. All participants reported that they had consumed starchy staples in the previous 24 h. Two hundred forty-five (57.9%) reported consuming dark green leafy vegetables, 72.8% vitamin A-rich fruits and vegetables, 74.9% other fruits and vegetables, and 76.4% legumes, nuts, and seeds. One-third (33.6%) consumed meat and fish, 29.1% egg, and 31.4% use milk and milk products. However, only 15.8% consumed organ meat in the 24 h preceding the survey. The mean IDDS score was 4.9 (SD 2.3). A bit lower than one third (30.3%) of study participants had inadequate dietary diversity as computed by less than four food groups, whereas 69.7% of them consumed adequate dietary diversity (Fig. 1).

### Nutritional status of peoples living with HIV

Overall, the prevalence of undernutrition was 29.3%, whereas 2.4% of study subjects were overweight. Of them, 28.9% and 29.6% of male and female participants were undernourished respectively. One-third (29.8%) of PLHIV within the age of 18–35 and 29.1% with the age of 36–55 were undernourished. Approximately, one-third (29.0%) of urban and 34.6% of rural residents were undernourished. More than half (56.5%) of PLHIV who had low dietary diversity were undernourished, but only 6.5% who had normal dietary diversity were undernourished. The mean BMI was 19.8 (SD = 2.4 kg/m2), whereas the median IDDS was 5(IQR = 3).

### Validity of minimum dietary diversity for women (MDD-W)

**Figure 2:**
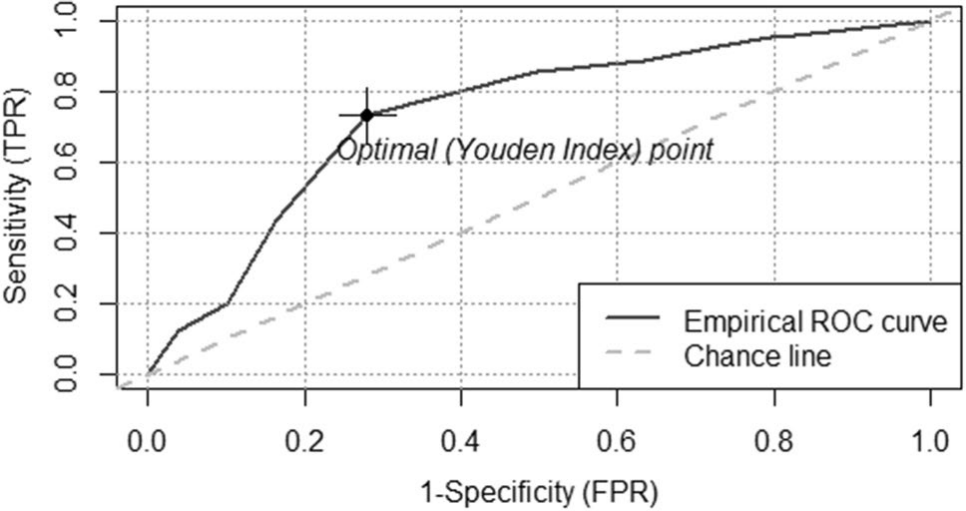
Receiver operating characteristic curve of MDD-W by using nutritional status as a benchmark of PLHIV in all Bahir Dar ART clinics, 2017^42^.

MDD-W is a good proxy indicator for measuring nutritional status of PLHIV with AUC = 74.1%, (95% CI = 68.5–79.6) (Fig. 2). The optimal cutoff point for MDD-W using nutritional status as a benchmark which maximized the Youden index was 4 (J = 0.45). The sensitivity and specificity of MDD-W for predicting low nutritional status with an optimal cut-off point of 4 were 73.0% (95%CI 65.0–81.0) and 72.0% (95%CI 66.0–77.0), respectively. Additionally, the Positive Predictive Value (PPV), Negative Predictive Value (NPV), Positive Likelihood Ratio (LR^+^), and Negative Likelihood Ratio (LR^−^) of MDD-W at optimal cut-off point were 53.0% (95%CI 45.0–61.0), 86.0% (95%CI 81.0–90.0), 2.62 (95%CI 2.12–3.24), and 0.37 (95%CI 0.27–0.50), respectively (Table 2).

**Table 2:** Sensitivity, specificity, PPV, NPV, LR^+^, and LR^−^ of MDD-W score among PLHIV in all ART clinics of Bahir Dar, 2017.

Cutoff	Sensitivity (95%CI)	Specificity (95%CI)	PPV (95%CI)	NPV (95%CI)	LR^+^ (95%CI)	LR^−^ (95%CI)	Youden Index
< = 1	0.12 (0.07, 0.19)	0.96 (0.93, 0.98)	0.58 (0.37, 0.77)	0.72 (0.67, 0.76)	3.18 (1.50, 6.72)	0.91 (0.85, 0.98)	0.08
< = 2	0.20 (0.13, 0.28)	0.90 (0.86, 0.93)	0.46 (0.33, 0.60)	0.72 (0.67, 0.77)	2.01 (1.23, 3.29)	0.89 (0.81, 0.98)	0.10
< = 3	0.44 (0.35, 0.53)	0.84 (0.79, 0.88)	0.53 (0.43, 0.63)	0.78 (0.73, 0.82)	2.68 (1.93, 3.72)	0.67 (0.57, 0.79)	0.28
< = 4	0.73 (0.65, 0.81)	0.72 (0.66, 0.77)	0.53 (0.45, 0.61)	0.86 (0.81, 0.90)	2.62 (2.12, 3.24)	0.37 (0.27, 0.50)	0.45
< = 5	0.85 (0.78, 0.91)	0.50 (0.44, 0.56)	0.42 (0.36, 0.49)	0.89 (0.83, 0.93)	1.72 (1.50, 1.97)	0.29 (0.19, 0.45)	0.35
< = 6	0.89 (0.82, 0.94)	0.37 (0.31, 0.43)	0.38 (0.32, 0.43)	0.88 (0.81, 0.93)	1.40 (1.26, 1.56)	0.31 (0.18, 0.52)	0.26
< = 7	0.93 (0.87, 0.97)	0.27 (0.22, 0.32)	0.35 (0.30, 0.41)	0.90 (0.81, 0.95)	1.27 (1.17, 1.38)	0.27 (0.14, 0.52)	0.24
< = 8	0.95 (0.90, 0.98)	0.20 (0.16, 0.26)	0.34 (0.29, 0.39)	0.91 (0.81, 0.97)	1.20 (1.11, 1.28)	0.24 (0.11, 0.53)	0.15
< = 9	0.96 (0.91, 0.99)	0.17 (0.13, 0.22)	0.33 (0.28, 0.38)	0.91 (0.80, 0.97)	1.16 (1.09, 1.24)	0.23 (0.10, 0.57)	0.13

### Internal consistency and validity of individual dietary diversity score

The reliability analysis showed that Cronbach's alpha value for all items of the individual dietary diversity measurement tool was 0.76. However, the item related to starchy stables had an item-total correlation of 0. When starchy stables items were deleted the Cronbach's Alpha increased to 0.78 and when legumes, nuts, and seeds were deleted the Cronbach's Alpha increased to 0.770 (Table 3).

**Table 3:** Reliability analysis result of individual dietary diversity measurement tool of PLHIV in all Bahir Dar ART clinics, 2017.

Items	Corrected item-total correlation	Cronbach's Alpha if Item deleted
Starchy stables	0.000	0.776
Vitamin-A rich vegetables and fruits	0.388	0.751
Other fruits and vegetables	0.407	0.747
Meat and fish	0.589	0.716
Dark green leafy vegetables	0.449	0.742
Organ meat	0.597	0.721
Egg	0.601	0.715
Legumes, nuts and seeds	0.256	0.770
Milk and milk product	0.564	0.721

**Figure 3:**
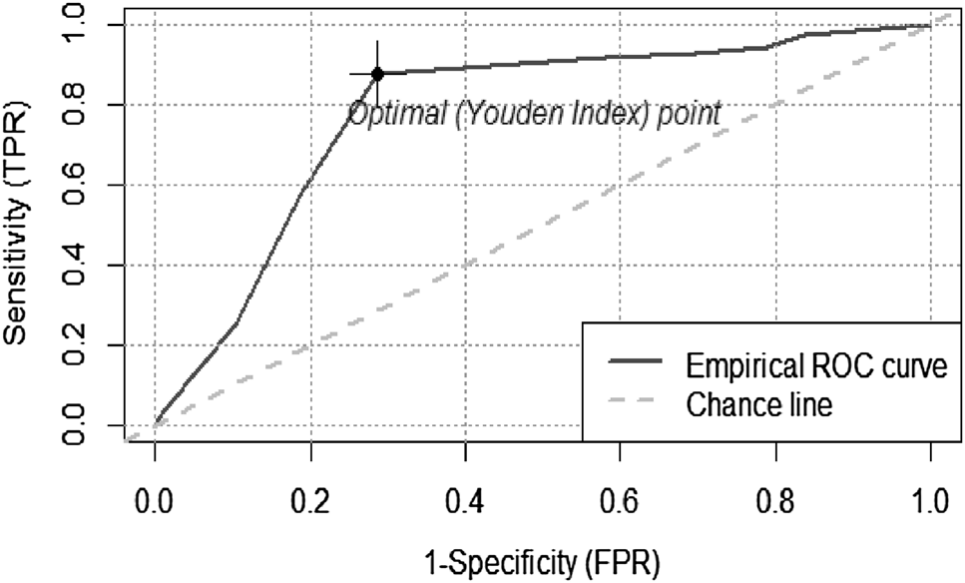
Receiver operating characteristic curve of IDDS by using nutritional status as a benchmark of PLHIV in all Bahir Dar ART clinics, 2017^42^.

IDDS is a good proxy indicator for measuring nutritional status of PLHIV with AUC = 78.5%, (95% CI = 73.9–83.4). The optimal cutoff point for IDDS using nutritional status as a benchmark that maximized the Youden index was 4 (J = 0.59) (Fig. 3). The sensitivity and specificity of IDDS for predicting low nutritional status with an optimal cut-off point of 4 and below were 88.0% (95%CI 81.0–93.0) and 71.0% (95%CI 66.0–76.0), respectively. Moreover, the Positive Predictive Value (PPV), Negative Predictive Value (NPV), Positive Likelihood Ratio (LR^+^), and Negative Likelihood Ratio (LR^−^) of IDDS at optimal cut-off four and below were 57.0% (95%CI 49.0–64.0), 93.0% (95%CI 89.0–96.0), 3.06 (95%CI 2.52–3.71), and 0.17 (95%CI 0.11–0.27), respectively (Table 4).

**Table 4:** Sensitivity, specificity, PPV, NPV, LR^+^, and LR^−^ of individual dietary diversity score among PLHIV in all ART clinics of Bahir Dar, 2017.

Cutoff	Sensitivity (95%CI)	Specificity (95%CI)	PPV (95%CI)	NPV (95%CI)	LR^+^ (95%CI)	LR^−^ (95%CI)	Youden Index
< = 1	0.04 (0.01–0.09)	0.99 (0.96–1.00)	0.56 (0.21–0.86)	0.71 (0.66–0.75)	2.91 (0.80–10.67)	0.97 (0.94–1.01)	0.03
< = 2	0.25 (0.18–0.34)	0.90 (0.86–0.93)	0.51 (0.38–0.64)	0.74 (0.69–0.78)	2.41 (1.53–3.80)	0.84 (0.75–0.93)	0.15
< = 3	0.57 (0.48–0.66)	0.81 (0.76–0.86)	0.57 (0.48–0.66)	0.82 (0.77–0.86)	3.06 (2.31–4.07)	0.53 (0.43–0.65)	0.39
< = 4	0.88 (0.81–0.93)	0.71 (0.66–0.76)	0.57 (0.49–0.64)	0.93 (0.89–0.96)	3.06 (2.52–3.71)	0.17 (0.11–0.27)	0.59
< = 5	0.92 (0.86–0.96)	0.42 (0.36–0.48)	0.40 (0.35–0.46)	0.92 (0.86–0.96)	1.58 (1.42–1.77)	0.19 (0.10–0.35)	0.34
< = 6	0.93 (0.87–0.97)	0.30 (0.25–0.36)	0.36 (0.31–0.42)	0.91 (0.83–0.96)	1.33 (1.22–1.46)	0.24 (0.12–0.46)	0.23
< = 7	0.94 (0.89–0.98)	0.21 (0.17–0.26)	0.34 (0.29–0.39)	0.90 (0.80–0.96)	1.20 (1.11–1.29)	0.27 (0.13–0.57)	0.15
< = 8	0.98 (0.93–0.99)	0.16 (0.12–0.21)	0.33 (0.28–0.38)	0.94 (0.83–0.99)	1.17 (1.10–1.23)	0.15 (0.05–0.47)	0.14

## Discussion

The prevalence of undernutrition in PLHIV was high (29.3%). Similarly, significant number of study subjects (30.3%) had inadequate dietary diversity. Majority (56.5%) of PLHIV who had low dietary diversity were undernourished. IDDS was a good reliable tool for measuring the dietary diversity of individuals. Its predictive power was good to classify the nutritional status of PLHIV. Additionally, the sensitivity and specificity of IDDS to correctly classify positive and negative outcomes were good. Among nine food groups, four were a cut-off point that maximizes the sensitivity and specificity of IDDS. Similarly, MDD-W has a comparable accuracy with IDDS for measuring nutritional status of PLHIV and its optimal cutoff point was four.

Majority PLHIV who had low dietary diversity were undernourished. This finding was consistent with several studies done in the world^45–47^. Inadequate dietary diversity contributes to micronutrient deficiency that lead to further HIV/AIDS progression and the reduction of CD4 count which increases risk of opportunistic infections^3^. This opportunistic infections altered nutrient intake, absorption and metabolism leading to malnutrition^3^.

In our study, the individual dietary diversity measurement tool showed good reliability (Cronbach's Alpha = 0.76). From nine items, starchy stables had no item-total correlation. Although it was inconsistent, it had no great effect on Cronbach's Alpha, if deleted it raises Cronbach's Alpha to 0.77. This might be due to all participants consumed this food group resulting in 0 variance. Therefore, it is better to retain this food group. Furthermore, legumes, nuts, and seeds had a fair item-total correlation (0.26) and only change Cronbach's Alpha to less than one unit, so this item was reliable for measuring the dietary diversity of PLHIV.

The overall accuracy of IDDS to correctly classify nutritional status measured with BMI was 78.5%. In LMIC, a relationship between dietary diversity scores and individuals’ nutritional status has already been shown in several studies^45,48,49^. However, validation of IDDS as a proxy indicator of nutritional status is limited. Our study is consistent with the study done in India which reported, individual dietary diversity score was a good proxy for the nutritional status of rural adults^25^. A study conducted in Sri Lanka adults and elders also showed a strong correlation between dietary diversity and BMI, indicating DDS are useful proxy indicators of nutrient adequacy^48,50^. The more varied and/or diversified the diet, as reflected by IDDS, the higher the anthropometric indices, reflecting a better nutritional status. Food intake increases when there is more variety in a meal or diet and later associated with increased body weight. On the other hand, inadequately diversified food leads to low micronutrient and caloric intake, which might contribute to the pathogenesis of HIV through increasing oxidative stress and compromised immunity and indirectly resulting in undernutrition^3^.

In this study, IDDS showed a good true positive and true negative rate in which 88.0% of undernourished PLHIV were correctly classified if they consumed less than four food group per day as a cutoff point from a total of nine food groups and 71.0% of well-nourished PLHIV were correctly classified by this cutoff point. This finding is inconsistent with a study done in Sri Lanka indicated that the best cutoff point for maximizing sensitivity and specificity of achieving 50% of MAR (mean adequacy ratio) was 4.5 for DDS^50^. Although we used a different benchmark, the cutoff point for optimal accuracy measures was similar. Hence, a cutoff point of 4 food groups could be taken to identify IDDS among PLHIV.

Our study found that MDD-W was a good proxy indicator for measuring nutritional status of PLHIV. This finding was supported by other studies which reported that dietary diversity was a good proxy indicator for micronutrient adequacy, dietary quality, and nutritional status of women^27,46,51,52^. MDD-W had a good sensitivity and specificity rate in which 73.0% of undernourished PLHIV were correctly classified if they consumed less than four food group per day as a cutoff point from a total of 10 food groups and 72.0% of well-nourished PLHIV were correctly classified by this cutoff point. This finding is in line with the study conducted on Burkina Faso^53^. Conversely, our result is inconsistent with several studies in which 4.5 and 5 were the optimal cutoff points that maximized the sensitivity and specificity of MDD-W^38,54–56^. This might be due to the difference in the reference variable and targeted population. Therefore, further largescale studies using different parameters in women of reproductive age group of PLHIV are needed to provide appropriate optimal cut-off point.

The present study provides evidence that IDDS and MDD-W have good sensitivity and specificity, implying that this tools are good for estimating the nutritional status of PLHIV as they measures the real dietary intake of PLHIV. Thus, IDDS and MDD-W can be used as a proxy indicator of nutritional status. However, they might not be used as a diagnostic or direct indicator for nutritional status. Hence, whenever equipment is available, we recommend using BMI as a measure of nutritional status. Whereas, in resource-limited settings IDDS and MDD-W can be used as a proxy indicator of an individual’s nutritional status.

This study has its strengths and limitations. The present study is the first in its type and can be used as a baseline for further research in the subject area. It is also interpreted considering the following limitations. Firstly, the data came from one geographical location, Bahir Dar. Therefore, using the standard cut-off point for this specific population might be not feasible for wider use, further validation studies are needed with larger sample sizes and in other locations. Then, it is not considered a repeated 24-h recall for measuring dietary diversity.

## Data Availability

It is available, based on a request we will give.

## Abbreviations

AIDS: Acquired immune deficiency syndrome
ART: Anti-Retroviral Therapy
AUC: Area Under ROC Curve
BMI: Body Mass Index
CI: Confidence Interval
HIV: Human Immune Deficiency Virus
IDDS: Individual Dietary Diversity Score
LR^−^: Negative Likelihood Ratio
LR^+^: Positive Likelihood Ratio
NPV: Negative Predictive Value
MPA: Mean Probability of Adequacy
PLHIV: People Living With Human Immune deficiency Virus
PPS: Proportional to Population Size
PPV: Positive Predictive Value
ROC: Receiver Operating Characteristic
SD: Standard Deviation
